# Impaired IgM Memory B Cell Function Is Common in Coeliac Disease but Conjugate Pneumococcal Vaccination Induces Robust Protective Immunity

**DOI:** 10.3390/vaccines12020214

**Published:** 2024-02-19

**Authors:** Olivia G. Moscatelli, Amy K. Russell, Lee M. Henneken, Melinda Y. Hardy, Nadia Mazarakis, Rachel Higgins, Jesse Ekin, Harry McLeod, Paul Simkin, Paul V. Licciardi, Vanessa L. Bryant, Jason A. Tye-Din

**Affiliations:** 1 Immunology Division, The Walter and Eliza Hall Institute, Parkville, VIC 3052, Australia; moscatelli.o@wehi.edu.au (O.G.M.);; 2 Department of Medical Biology, University of Melbourne, Parkville, VIC 3052, Australia; 3 Department of Gastroenterology, The Royal Melbourne Hospital, Parkville, VIC 3052, Australia; 4 The Murdoch Children’s Research Institute, Parkville, VIC 3052, Australia; 5 Department of Radiology, The Royal Melbourne Hospital, Parkville, VIC 3052, Australia; 6 Department of Paediatrics, The University of Melbourne, Parkville, VIC 3052, Australia; 7 Department of Clinical Immunology, The Royal Melbourne Hospital, Parkville, VIC 3052, Australia

**Keywords:** coeliac disease, IgM memory B cells, hyposplenism, pneumococcal vaccination

## Abstract

Coeliac disease (CD) is associated with hyposplenism, an acquired impairment of spleen function associated with reduced IgM memory B cells and increased susceptibility to serious pneumococcal infection. Little is known about the immune implications of hyposplenism in CD or the optimal pneumococcal vaccination strategy. In this study, the immune effects of hyposplenism in CD, and the accuracy of screening approaches and protective responses induced by two different pneumococcal vaccines were examined. Active and treated CD cohorts, and healthy and surgically splenectomised controls underwent testing for the presence of Howell–Jolly bodies and pitted red cells, spleen ultrasound, and immune assessment of IgM memory B cell frequency and IgM memory B cell responses to T cell-dependent (TD) or T cell-independent (TI) stimulation. Responses following conjugate (TD) and polysaccharide (TI) pneumococcal vaccination were compared using ELISA and opsonophagocytic assays. Although hyposplenism is rare in treated CD (5.1%), functional B cell defects are common (28–61%) and are not detected by current clinical tests. Conjugate pneumococcal vaccination induced superior and sustained protection against clinically relevant serotypes. Clinical practice guidelines in CD should recommend routine pneumococcal vaccination, ideally with a conjugate vaccine, of all patients in lieu of hyposplenism screening.

## 1. Introduction

Coeliac disease (CD) is a common immune illness triggered by dietary gluten with an estimated global seroprevalence of 1.4% [1]. The typical enteropathy of CD is mediated by pro-inflammatory intestinal HLA-restricted CD4^+^ T cells responding to specific gluten peptides, with gut plasma cells implicated as the major antigen-presenting cell [2,3,4]. In this context, B cells provide help to gluten-specific T cells to amplify the pathogenic response to gluten. Although primarily a small intestinal disease, the systemic inflammatory response of CD leads to extraintestinal complications such as reduced bone density and an increased risk of lymphoproliferative malignancy [5]. One of these, hyposplenism, an acquired impairment of spleen function [6,7,8,9], is reported in 12–80% [10,11,12,13] of CD sufferers, especially those with autoimmunity, refractory CD (non-response to treatment with a gluten-free diet; GFD), or those diagnosed after 50 years of age [10].

A concern with hyposplenism is the increased susceptibility to serious infections with encapsulated bacteria [14,15,16,17,18,19], most commonly *Streptococcus pneumoniae* (pneumococcus). Large population studies have shown that the increased risk of pneumococcal sepsis contributes to elevated morbidity and mortality in CD. The excess risk of pneumococcal infection in hospitalised CD patients compared to the general population is significant (HR = 3.90, 95% CI 2.20–7.00) [19]. Screening for hyposplenism assesses the spleen’s filtering function, with impairment indicated by the presence of Howell–Jolly bodies (HJBs), nuclear inclusion remnants in red blood cells [20,21], or pitted red cells (PRCs) resulting from the accumulation of membrane vacuoles [22]. The immunodeficiency linked to hyposplenism is attributed to reduced numbers of circulating IgM memory B cells. These cells require intact spleen function for their development and survival, unlike switched memory B cells, which are present at normal frequencies in asplenic patients [23,24,25,26]. IgM memory B cells are critical for protective responses to T cell-independent (TI) stimuli, particularly encapsulated bacteria [23,27,28,29]. While a reduced IgM memory B cell population is associated with hyposplenism in CD [10,24,25], their function in CD has not been investigated. Furthermore, it remains uncertain whether screening for hyposplenism based on HJBs or PRCs is sufficiently sensitive to identify patients with suboptimal spleen function who remain at risk of pneumococcal sepsis.

While pneumococcal vaccination is recommended for patients with asplenia or hyposplenism with HJBs present [30,31,32], there is no consensus recommendation for vaccination in CD [33,34,35]. Two frequently employed pneumococcal vaccines are a 23-valent pneumococcal capsular polysaccharide formulation (23vPPV) encompassing serotypes 1, 2, 3, 4, 5, 6B, 7F, 8, 9N, 9V, 10A, 11A, 12F, 14, 15B, 17F, 18C, 19F, 20, 22F, 23F, and 33F, and a 13-valent pneumococcal conjugate vaccine (13vPCV) encompassing serotypes 1, 3, 4, 5, 6A, 6B, 7F, 9V, 14, 18C, 19A, 19F, and 23F. The polysaccharide formulation of 23vPPV leads to the direct activation of B cells independent of T cells (TI), which induces anti-capsular IgG antibodies as well as short-lived IgM+ plasmablasts, but does not typically generate a long-lived memory B cell response [27]. The serotype-specific IgG response to 23vPPV is an important test used in the investigation of suspected immune deficiency. In contrast to 23vPPV, the protein-conjugated 13vPCV induces T cell-dependent (TD) responses, which generates higher avidity antibodies and immunologic memory [36]. As PCV13 generates the formation of both serotype-specific antibodies and memory B cells, it is associated with a longer duration of vaccine-induced protection. Protective immunity following 13vPCV is more effective in splenectomised patients [37]. Notably, the comparative immunogenicity of 23vPPV and 13vPCV in CD, and the effect of IgM memory B cell deficits in this population, have not been assessed. These data are needed to inform vaccination recommendations.

Here, we examined hyposplenism in active and treated CD using traditional clinical readouts coupled with immune phenotyping and functional studies. We assessed the impact of the GFD on hyposplenism and the immune consequences of alterations in IgM memory B cell frequency and function. We also conducted a randomised vaccination study of 23vPPV and 13vPCV to compare protective immunity and its relationship to pre-existing immune defects. Our findings provide a rational basis for managing hyposplenism in CD and preventing serious pneumococcal disease.

## 2. Materials and Methods

### 2.1. Participants

Participants were recruited using a local CD Research Database and via advertisements placed to members of patient support association Coeliac Australia. CD was confirmed based on consistent small intestinal histology showing villous atrophy and positive CD serology. Two cohorts of CD participants were recruited, one within 4 weeks of diagnosis and the initiation of a GFD (active CD), and a separate cohort with established disease on a GFD for at least 12 months (treated CD). Control cohorts comprised healthy volunteers without CD (confirmed by negative CD serology whilst eating gluten) and participants who had previously undergone surgical splenectomy (splenectomised controls, SP).

### 2.2. Clinical and Hyposplenism Assessment

Blood (50–150 mL) was collected from all participants, and repeat samples collected from active CD patients after 6 months on a GFD. Serum was assessed for transglutaminase-IgA, deamidated gliadin peptide-IgG, total complement (C3, C4), and total IgA, IgG, and IgM levels. If unknown, HLA-DQ2/DQ8 genotype was determined using the SNP polymorphism tagging method [38], and if the imputed HLA remained unresolved, complete HLA genotyping via PCR was performed. Hyposplenism screens were performed by a clinical haematologist using a peripheral blood smear examining for HJBs, and via the assessment of PRCs using the method established by Corazza et al. [22]. Manual counts of PRCs utilising differential interference contrast microscopy were based on the mean count across 5 high powered fields, and were the mean of two trained independent readers.

Spleen ultrasound was performed, and images assessed by a specialist radiologist. To express spleen volume, the splenic index was calculated using the standard prolate ellipsoid formula (length × width × depth × 0.52) as frequently used for the volume estimation of irregularly shaped organs [39].

### 2.3. Flow Cytometry, Antibodies, and Reagents

Peripheral blood mononuclear cells (PBMCs) were isolated from whole blood samples using density gradient centrifugation (Leucosep, Interpath Services, Somerton, VIC, Australia) and cryopreserved. PBMCs were immunophenotyped to determine proportions of B cell subsets. Anti-mouse compensation beads (eBioscience, San Diego, CA, USA) were used as single colour controls for compensation analysis, and Fluorogold to exclude dead cells (Invitrogen, Carlsbad, CA, USA). All phenotypic data were acquired on a Fortessa X-20 using BD FACSDiva 8.0.1 software, and all acquired flow data analysed using FlowJo 10.7.1. The following antibodies were used: anti-CD10 (HI10a; PE-CF594), anti-CD20 (2H7; BUV395), anti-CD21 (B-ly4; APC), anti-CD24 (ML5; BV605), anti-CD38 (HIT2; PerCP-Cy5.5), anti-IgG (G18-145; PE-Cy7), and biotinylated anti-IgA/G/M (BD Biosciences, Franklin Lakes, NJ, USA); anti-CD27 (M-T271; FITC), anti-IgA (IS11-8E10; PE), anti-IgD (IA6-2; BV510), and anti-IgM (SA-DA4; EF450) (Miltenyi Biotech, Gladbach, Germany); anti-IgM (MHM-88; AF647) (Biolegend, San Diego, CA, USA); goat anti-human IgA/G/M, and streptavidin-HRP (Southern Biotech, Birmingham, AL, USA); and IgA/G/M standard (Sigma-Aldrich, Saint Louis, MO, USA).

### 2.4. Functional B Cell Assays

B cells were enriched from cryo-preserved PBMCs using the EasySep Human B Cell Isolation Kit (Stem Cell). Naïve, isotype-switched, and IgM-expressing memory B cells were isolated by sorting CD20+CD27−IgG/A−, CD20+CD27+IgG/A+IgM−, and CD20+CD27+IgG/A−IgM+, respectively [40,41,42,43,44], using an Aria Fusion (Becton Dickinson, Franklin Lakes, NJ, USA) with purity > 97%. Purified B cell populations were cultured at 37 °C with 5% CO_2_ in B cell media (as previously described [45]) with T-dependent (TD) stimuli CD40L (200 ng/mL; Enzo Life Science, New York, NY, USA) and IL-21 (50 ng/mL; Peprotech, Cranbury, NJ, USA) [46,47,48,49,50], or labelled with the division tracking dye, Cell Trace Violet (CTV; Thermofisher, Waltham, MA, USA), and cultured with T-independent (TI) stimuli anti-human IgA/G/M (α-Ig; 200 μM; Jackson Immunoresearch, West Grove, PA, USA) and CpG (50 μg/mL; Miltenyi Biotech, Tokyo, Japan) [51,52]. B cells were cultured in 96-well plates for 3–5 days (25 × 104 cells/200 μL/well; Corning, New York, NY, USA).

### 2.5. Assessing B Cell Function

Phenotypic analysis: In vitro activated naïve, switched-, and IgM- memory B cells were harvested after 5 days (TD-stimulated) or after 3, 4, and 5 days (TI-stimulated), incubated with an antibody cocktail, and acquired on a Fortessa X-20. The absolute number of TI-stimulated cells was calculated by adding a known number of CaliBRITE beads (BD Biosciences, Franklin Lakes, NJ, USA) to each sample before staining [44].

Enzyme-Linked Immunosorbent Assay (ELISA): The secretion of IgM, IgG, and IgA was determined using Ig H chain-specific immunoassays, as previously [42,44].

### 2.6. Pneumococcal Vaccination and Assessment of Responses

Thirty-five pneumococcal-vaccination-naïve treated CD participants were randomised to receive 23vPPV (Pneumovax23) or 13vPCV vaccination (Prevenar13). Serum samples were collected at baseline, 4 weeks, and 6 months post vaccination.

ELISA: IgG concentration specific to 13 serotypes common to Pneumovax23 and Prevenar13 was determined using a modified version of the gold standard World Health Organisation ELISA [53].

Multiplexed opsonophagocytic assay (MOPAs): Functional antibody responses against serotypes 1, 5, 6B, 7F, 14, 18C, 19A, and 23F were measured using HL-60 cell line-derived granulocytes as effector cells. Opsonisation indices were calculated as the serum dilution required to kill 50% of pneumococcus bacteria [54].

### 2.7. Powering

The study was powered to detect changes in IgM memory B-cell frequency between cohorts. Based on a prior similar study [10] and where healthy data with SD were available [24], assuming that mean IgM memory B-cell frequency is 8.9% in the patient cohort and 15% in healthy controls (SD = 6.9), an alpha value of 0.05, enrolment ratio of 2, and 80% power will be achieved with 15 healthy volunteers and 30 CD cases. As IgM memory B-cell frequency has been reported to be as low as 3.4% in CD [25], our sample size should support robust powering.

### 2.8. Statistical Analysis

Data were analysed using Prism software (GraphPad 9.4.1) and compared between cohorts using Mann–Whitney U-tests (two-tailed); correlations were examined using Spearman’s rank correlation test. Pneumococcal serotype-specific antibody concentrations were log (base 10) transformed and compared between vaccines by two-tailed *t*-tests. *p* values < 0.05 were considered significant.

## 3. Results

### 3.1. Overt Hyposplenism Is Uncommon in CD

The frequency of hyposplenism as defined by HJBs present on a blood film and/or >4% PRCs (as previously defined [20,22]) was determined in each cohort (Table 1). All splenectomised patients met this criterion with the exception of a single patient who was subsequently excluded from analysis on suspicion of residual splenic function. Within the CD cohort, hyposplenism was detected in three treated CD (3/59; 5.1%) and one active CD participant (1/6; 17%). While there was complete concordance of results between HJBs and PRCs within the splenectomised cohort, two CD individuals with demonstrable HJBs had normal PRC values (Supplementary Table S1).

The impact of complicated CD (concurrent autoimmunity and/or refractory CD) and age at CD diagnosis was assessed (Table 2). A higher frequency of hyposplenism was observed in cases of complicated CD (1/12, 8.3%) and in patients diagnosed after 50 years (2/18, 11.1%), which together accounted for 2/3 of hyposplenic treated CD cases. Hyposplenism in treated CD without complications or late-onset diagnosis occurred in only 2.9% (1/35). Despite increased frequencies, no significant differences were detected in PRC value or spleen volume between these groups. Spleen volume was diminished in three treated CD patients: two males (51 cm^3^ and 25 cm^3^; 5th–95th percentile 90–334 cm^3^), one of whom was diagnosed after 50 years, and one female (33 cm^3^, 5th–95th percentile 64–231 cm^3^) with detectable HJBs, elevated PRCs (41.3%), and concurrent autoimmunity. Aside from these cases, spleen size in treated and active CD cohorts remained comparable to historical data from healthy cohorts [39]. There were no correlations between HJBs, PRCs, and spleen size. Collectively, overt hyposplenism is infrequent in treated CD but is more common in patients with active disease, a later age of diagnosis, concurrent autoimmunity, and/or refractory CD.

### 3.2. IgM Memory B-Cell Frequency Is Not Reduced in CD

**Figure 1:**
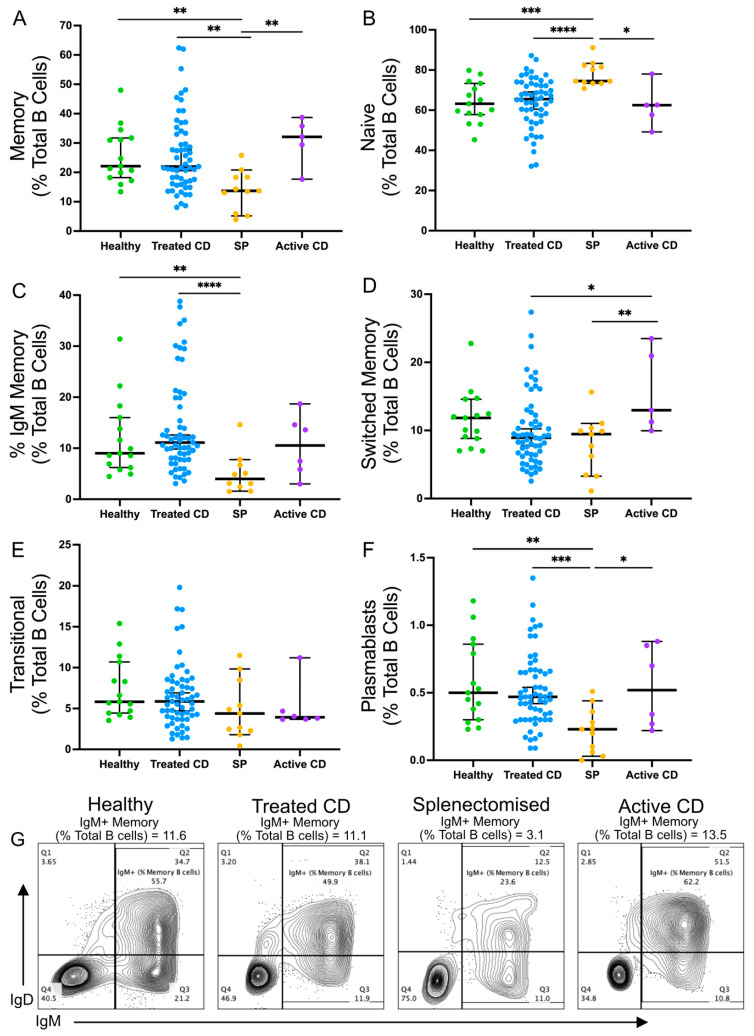
Frequencies of B cell populations in healthy, treated CD, active CD, and splenectomised cohorts. The frequencies of the following B cell populations are shown for each cohort as a proportion of the total B cell population: memory B cells ((**A**); CD20+CD27+CD10−), naïve B cells ((**B**); CD20+CD27−CD10−), IgM memory B cells ((**C**); CD20+CD10−CD27+IgM+IgG−IgA−), switched memory B cells ((**D**); CD20+CD10−CD27+IgM−IgG+IgA+), transitional B cells ((**E**); CD20+CD10+CD27−), and plasmablasts ((**F**); CD20+CD27hiCD38+). Representative flow plots from each cohort show memory B cells with an IgM+ gate (**G**). Frequencies are compared using Mann–Whitney U-tests (two-tailed). Horizontal lines on graphs indicate median, and error bars indicate 95% confidence intervals. * *p* < 0.05, ** *p* < 0.01, *** *p* < 0.001, and **** *p* < 0.0001.

The frequencies of naïve, transitional, memory, isotype-switched memory, IgM-expressing memory B cells, and plasmablast populations were analysed [41] (Figure 1). A lower frequency of IgM memory B cells was observed in splenectomised individuals compared to healthy controls consistent with previous findings (median, 4.98% compared to 9.25%; *p* = 0.0023) [26]. In the active and treated CD cohorts, all B-cell frequencies were comparable to healthy controls. Similarly, there were no differences in IgM memory B-cell frequency between complicated and uncomplicated CD or between patients diagnosed before or after 50 years.

### 3.3. Reduced IgM Memory TD Responses in Treated CD Patients

**Figure 2:**
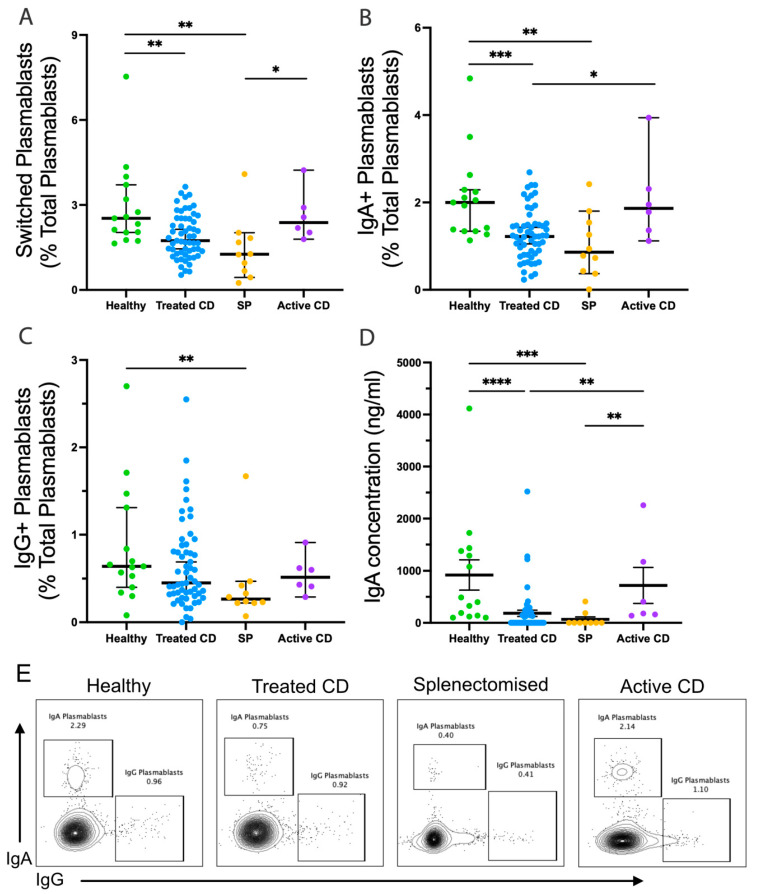
IgM memory B cells responses following TD stimulation. Frequencies of switched plasmablasts ((**A**); CD20+CD27hiCD38+IgA+/IgG+), IgA+ plasmablasts ((**B**); CD20+CD27hiCD38+IgA+IgG−), and IgG+ plasmablasts ((**C**); CD20+CD27hiCD38+IgA−IgG+) are shown as a proportion of total plasmablasts (CD20+CD27hiCD38+). Secreted IgA concentration (ng/mL) is shown (**D**). Representative flow plots show the frequency of IgA and IgG plasmablasts from the healthy, treated CD, and splenectomised (SP) cohort (**E**). Frequencies are compared using Mann–Whitney U-tests (two-tailed). Horizontal lines on graphs indicate median, and error bars indicate 95% confidence intervals. * *p* < 0.05, ** *p* < 0.01, *** *p* < 0.001, and **** *p* < 0.0001.

While the frequency of IgM memory B cells has been assessed in CD [10], their function has not. Naïve, isotype-switched memory, and IgM-expressing memory B-cell responses were examined in each cohort after TD stimulation by assessing their capacity for isotype switching and their potential to differentiate into antibody secreting plasmablasts (Figure 2). There were no differences in naïve or isotype-switched memory B-cell responses to TD stimulation between cohorts. In the treated CD cohort, the TD stimulation of IgM memory B cells induced the generation of fewer switched plasmablasts compared to the healthy cohort (median, 1.74% and 2.53%, respectively; *p* = 0.0029), with 40% (23/57) of treated CD responses below the healthy range (Figure 2A). Strikingly, this reduction in the treated CD cohort closely paralleled observations in splenectomised individuals (median, 1.26%; *p* = 0.0015). This reduction was largely due to the decreased generation of IgA-expressing plasmablasts, with 42% (24/57) of treated CD responses below the healthy range and almost a 2-fold difference between the medians (1.22% and 2%, respectively; *p* = 0.0006; Figure 2B).

This was validated by reduced concentrations of secreted IgA, with a 5-fold difference in the medians between treated CD and healthy cohorts (183.9 ng/mL, and 920.4 ng/mL, respectively; *p* < 0.0001; Figure 2D). Notably, these values were below the healthy range and undetectable in 59% of treated CD cases. However, no differences were observed in the ability to undergo isotype switching and differentiation to IgG-expressing plasmablasts (Figure 2C).

Age at diagnosis and the presence of comorbidities did not affect the frequency of switched plasmablasts; however, there was a positive correlation with the duration of adherence to a GFD in treated CD (R = 0.295, *p* = 0.032), suggesting gluten exposure and/or disease duration impact spleen function. Taken together, there is a significantly reduced capacity of IgM memory B cells to undergo isotype-switching following TD stimulation in CD, approaching that seen in splenectomised individuals.

### 3.4. Impaired IgM Secretion in TI-Stimulated IgM Memory B Cells from Treated CD Patients

**Figure 3:**
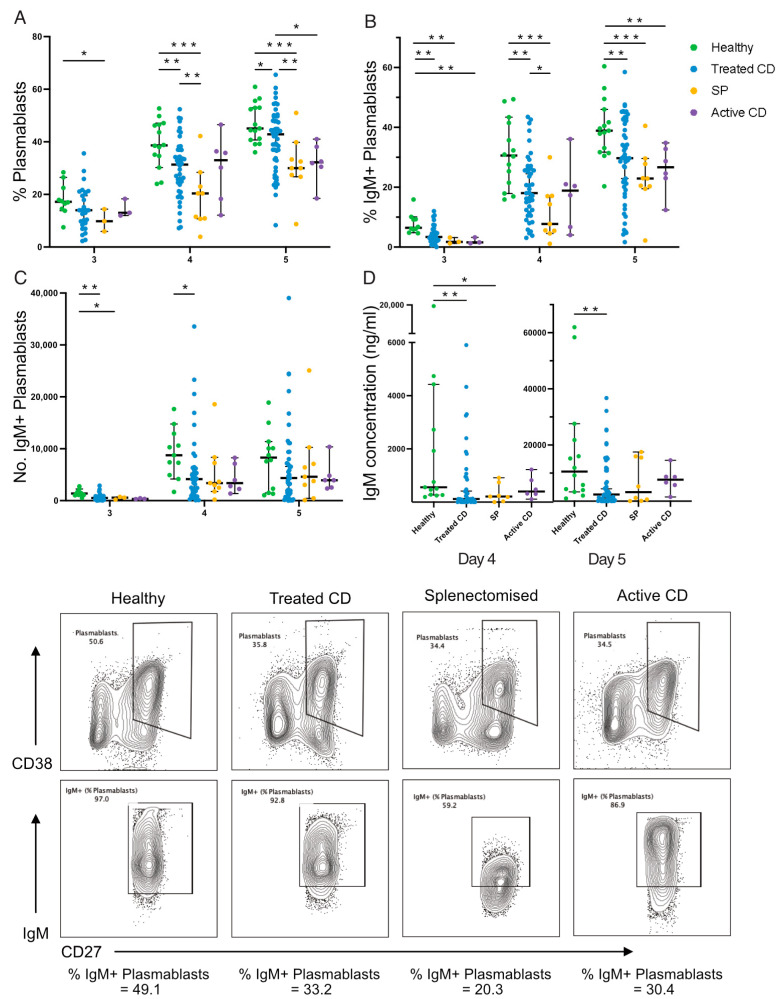
IgM+ plasmablast frequency and function following TI stimulation. The frequency of plasmablasts (**A**), frequency (**B**) and absolute number (**C**) of IgM+ plasmablasts, and secreted IgM concentration (ng/mL; (**D**)) are shown. Representative flow plots show the gating of plasmablasts (CD38+CD27hi) and IgM+ plasmablasts for each cohort: healthy, treated CD, splenectomised (SP), and active CD. Frequencies are compared using Mann–Whitney U-tests (two-tailed). Horizontal lines on graphs indicate median, and error bars indicate 95% confidence intervals. * *p* < 0.05, ** *p* < 0.01, and *** *p* < 0.001.

Next, IgM memory B-cell responses to TI stimulation were examined (Figure 3). IgM memory B cells isolated from active CD, treated CD, and splenectomised cohorts demonstrated significantly reduced differentiation into antibody-secreting plasmablasts compared to healthy donors after 4 and 5 days (Figure 3A). This decreased differentiation potential was significantly more pronounced in cultures from splenectomised individuals, compared to the treated CD group (*p* < 0.01). The frequency of IgM+ plasmablasts generated fell below the healthy range in 61% (17/28) of treated CD on day 3, in 38% (17/44) on day 4, and in 28% (15/54) on day 5 (Figure 3B). Again, this reduction was greater in splenectomised participants than treated CD, but only on day 4 (median, 7.73% vs. 18.05%; *p* = 0.03). This reduction in frequency corresponded to a lower absolute count of IgM+ plasmablasts in the treated CD and splenectomised cohorts compared to healthy controls on day 3 (median, 524, 572, and 1393; *p* = 0.001, *p* = 0.03, respectively) and day 4 (median, 4150, 3382, and 8738; *p* = 0.02, *p* = 0.06, respectively; Figure 3C), with these populations more than halved in treated CD participants.

IgM concentration was below the healthy range in 46% (18/39) of treated CD responses on day 4 (median, 556 ng/mL and 19 ng/mL, respectively; *p* = 0.005) and 33% (16/48) on day 5 (median, 10,546 ng/mL and 2386 ng/mL, respectively; *p* = 0.004; Figure 3D), further supporting the differentiation defect. The examination of division kinetics via CTV-division tracking revealed no differences in the frequency of cells in each division or in the frequency of undivided cells (Supplementary Figure S1). Collectively, the responses of IgM memory B cells to TI stimulation is impaired in CD similar to that observed in splenectomised individuals but to a lesser extent.

### 3.5. Spleen Function Is Not Impacted by Coeliac Disease Activity

**Figure 4:**
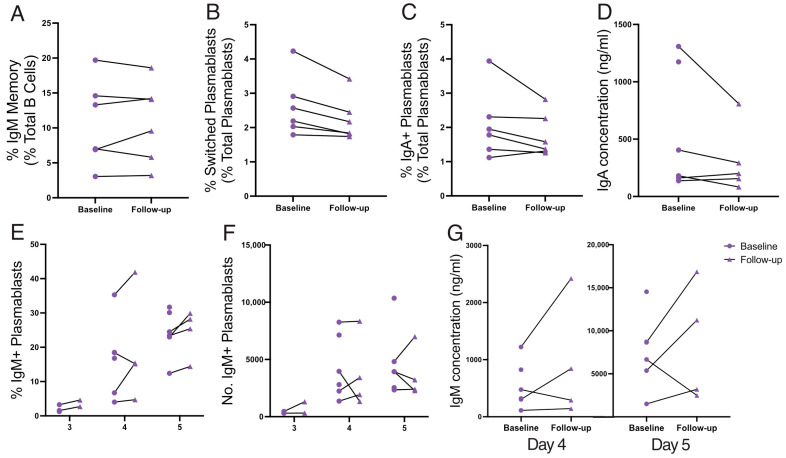
Spleen function from active disease (baseline) to follow-up at 6 months on a GFD. IgM memory B-cell frequency is shown (**A**). Sort-purified IgM memory B cells after TD stimulation: frequencies of switched plasmablasts (**B**) and IgA+ plasmablasts (**C**), and secreted IgA concentration (ng/mL; (**D**)). Sort-purified IgM memory B cells after TI stimulation: frequency (**E**) and absolute number (**F**) of IgM+ plasmablasts, and IgM concentration (ng/mL; (**G**)). Frequencies are compared using Wilcoxon matched-pairs tests (two-tailed).

Limited data exist regarding the impact of a GFD on spleen function, with previous studies primarily focused on spleen filtering capacity [11,12,55,56]. To address this gap, immunophenotyping and functional studies were repeated in patients after a 6-month adherence to a strict GFD (Figure 4). No differences were observed in the frequency of PRCs or IgM memory B cells between baseline (active CD) and 6-month follow-up (Supplementary Table S1; Figure 4A). Similarly, there were no consistent improvements in IgM memory B-cell responses to TD or TI stimulation (Figure 4B–G). Therefore, spleen function is not changed by 6 months of a GFD.

### 3.6. HJBs, PRCs, and Spleen Size Can Detect Immune Defects but Lack Sensitivity

Treated CD patients classified as hyposplenic based on HJBs, PRCs, or small spleen size were analysed to examine the association with in vitro IgM memory B-cell defects (Table 3). All hyposplenic patients showed responses below the healthy range for at least one functional B-cell metric. Based on the high frequency of immune defects demonstrated above and the high proportion that were not accompanied by these traditional measures of hyposplenism, the findings suggest that HJBs, PRCs, and spleen size are relatively insensitive measures of spleen function.

### 3.7. Conjugate Vaccine Induced a Higher Protective Response to Pneumococcus

The protective antibody response to both polysaccharide (23vPPV; Pneumovax23) and conjugate (13vPCV; Prevenar13) vaccines was assessed next. Pneumococcal-vaccination-naïve treated CD participants were randomised to receive 23vPPV (n = 19) or 13vPCV (n = 16). All participants tolerated vaccination well, with no reports of serious adverse events (Supplementary Table S2).

**Figure 5:**
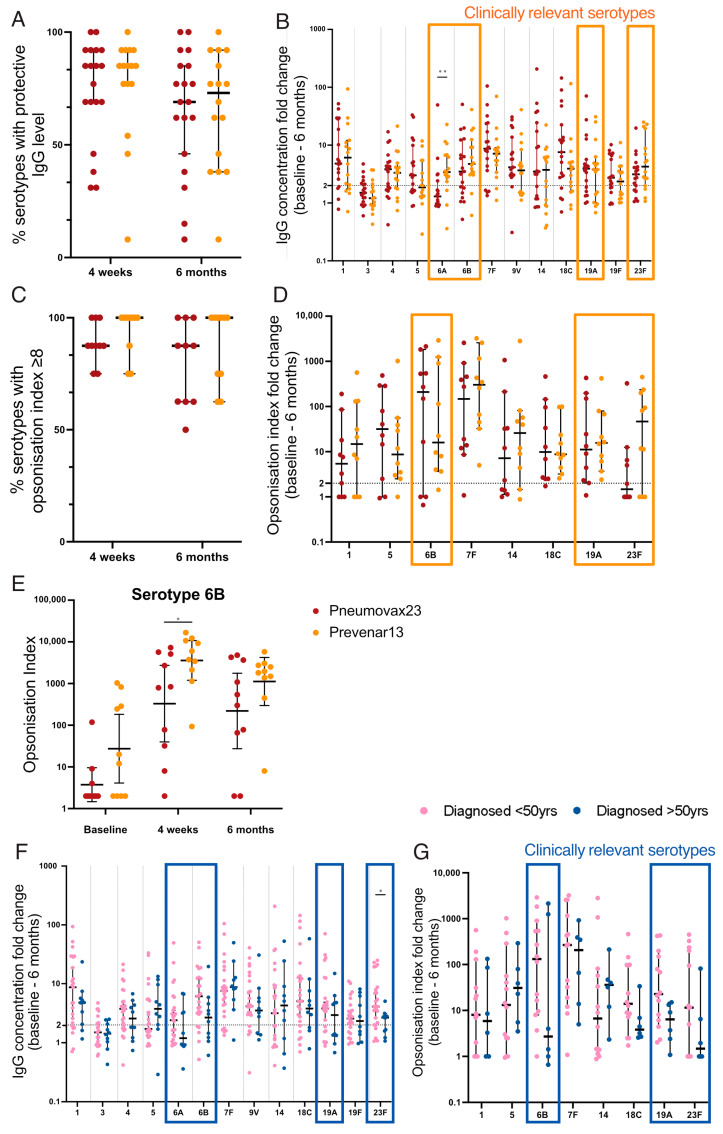
Antibody titre and opsonisation index after pneumococcal vaccination in CD patients. Each data point represents the proportion of serotypes with protective IgG concentrations ((**A**); ≥2 fold-change) or opsonisation index ((**C**); OI ≥ 8). Fold change from baseline to 6 months post vaccination is shown for IgG concentration (**B**) and opsonisation index (**D**); protective threshold ≥2-fold change (indicated by dashed line); clinically relevant serotypes indicated by orange boxes. Opsonisation index is shown for serotype 6B at all three timepoints (**E**). (**F**,**G**) Treated CD patients stratified by age at diagnosis. Fold change from baseline to 6 months post vaccination is shown for IgG concentration (**F**) and opsonisation index (**G**); clinically relevant serotypes indicated by blue boxes. Mann–Whitney U-tests were performed to compare fold changes. Horizontal lines indicate median, and error bars indicate 95% confidence intervals. Pneumococcal serotype-specific antibody concentrations were log (base 10) transformed and compared between vaccines using two-tailed *t*-tests (**E**). * *p* < 0.05, ** *p* < 0.01.

Serotype-specific serum IgG levels were measured, with a ≥2-fold increase considered protective [57]. At 4 weeks post vaccination, the median proportion of serotypes, out of the 13 common to both vaccines, for which each individual demonstrated protective IgG levels was 85% (11/13) for both 23vPPV and 13vPCV. By 6 months post vaccination, the proportions declined to 69% (9/13) for 23vPPV and 73% (9.5/13) for 13vPCV (Figure 5A). Protective responses to serotypes 6A, 6B, 19F, and 23F were a focus, as defective immunity to these serotypes is associated with a higher risk of pneumonia and mortality [58], and thus they are considered the most clinically relevant serotypes for protection. The fold change in IgG concentration from baseline to 6 months post vaccination to these clinically significant serotypes was higher following 13vPCV vaccination compared to 23vPPV, with a significant difference for serotype 6A (median, 3.09 compared to 1.3; *p* = 0.005; Figure 5B). Both vaccines failed to induce protective responses to serotype 3, consistent with previous findings [59].

Next, the functional antibody response was measured using an opsonophagocytic assay to determine the ability of serotype-specific antibodies to opsonise and promote the phagocytosis of pneumococcus. An opsonisation index (OI) ≥ 8 was considered protective [57]. The median proportion of serotypes for which each individual demonstrated protective OI was 88% (7/8) for 23vPPV and 100% (8/8) for 13vPCV at both 4 weeks and 6 months post vaccination (Figure 5C). The fold change in OI from baseline to 6 months was higher following 13vPCV vaccination than 23vPPV for clinically relevant serotypes, 19F (median, 15.65 compared to 11.15; *p* = 0.94) and 23F (median, 46.55 compared to 1.48; *p* = 0.76; Figure 5D). Notably, the OI for serotype 6B was >10-fold higher following 13vPCV vaccination at 4 weeks (mean, 3585.5 compared to 329.5; *p* = 0.02; Figure 5E) and 6 months (mean, 1120.5 compared to 220.4) post vaccination compared to 23vPPV. However, the higher response was not reflected in the fold change from baseline to 6 months post vaccination due to the high baseline OI for 13vPCV compared to the 23vPPV-vaccinated cohort (mean, 27.31 compared to 3.74; *p* = 0.07). Importantly, the frequency of participants demonstrating protective OI responses against serotypes 6B and 23F was 20% higher following 13vPCV vaccination. In combination, 13vPCV generates higher and more sustained responses to clinically significant pneumococcal serotypes in CD.

### 3.8. Impaired Vaccine Responses in Those Diagnosed after 50 Years of Age

Pneumococcal vaccine responses in treated CD patients diagnosed after 50 years were examined given their high rate of functional B-cell defects (Figure 5F,G). There were trends towards lower antibody responses in this cohort. Importantly, this was evident in all clinically relevant serotypes and a significant reduction in the fold change of IgG concentration from baseline to 6 months post vaccination was observed for serotype 23F (median, 2.7 compared to 4.1; *p* = 0.028; Figure 5F). These data raise concerns about the potential impaired vaccine responses in patients diagnosed at an older age who are more vulnerable to severe pneumococcal infection.

## 4. Discussion

Pneumococcal sepsis is an important cause of morbidity and mortality in CD, but little attention has been given to understanding defective immunity to this bacterium and how this can inform vaccination recommendations in CD. Here, we found that hyposplenism defined by traditional readouts is less common than earlier reports [10,12,56], but that subtle functional immune B-cell defects are common. Consistent with the lower rate of overt hyposplenism, the frequency of IgM memory B cells was normal in treated and active CD. While our findings contrast with historical CD data published almost two decades ago [10,25], the median IgM memory B-cell frequencies in our healthy (9.25%) and splenectomised (4.98%) cohorts were highly concordant with another Australian study (approximately 9% and 4.5%, respectively) [26]. Notably, our contemporary cohort includes many CD patients with minimal or no symptoms and limited comorbidities, in contrast to historical studies that more frequently included patients with severe clinical phenotypes expected to have higher rates of complications, including hyposplenism. Although we identified a lower rate of overt hyposplenism, our results support those of Di Sabatino and colleagues who showed higher rates of hyposplenism in CD complicated by autoimmune diseases, refractory CD, and a later age of diagnosis [10].

This study provides novel insights into the functional immune implications of hyposplenism. A striking finding is that subtle functional defects in IgM memory B cells are present and common even in well-treated CD patients following a strict GFD diet who have negative CD serology. These defects affect up to 61% of patients and include impaired isotype-switching following TD stimulation and impaired differentiation and IgM production following TI stimulation. This supports the notion that hyposplenism is characterised by functional immune defects similar to asplenia, although to a milder degree.

These findings have implications for the management of hyposplenism in CD and provide additional insights into the mechanistic basis for increased susceptibility to sepsis from encapsulated bacteria in both CD and splenectomised individuals. IgM+ plasmablasts are responsible for rapidly producing large amounts of IgM to opsonise bacteria for destruction by phagocytosis [28,29]. Thus, lower IgM+ plasmablasts and IgM secretion could result in decreased bacterial clearance early in the infection, leading to higher bacterial loads that are more likely to overrun the immune system and cause sepsis. However, the underlying mechanism for this dysfunction in CD and other gastrointestinal diseases such as inflammatory bowel disease [24] remains unknown [60].

Despite the high prevalence of these immune defects, they were largely undetected by standard clinical tests. Whilst all patients classified as hyposplenic by HJBs, PRCs, or spleen size had defects in the TD and TI IgM memory B-cell responses, this accounted for a very small proportion of CD patients. As noted by others, the clinical implementation of the PRC approach is not straightforward as it requires a specialised microscope, the technique is time-consuming, and findings are susceptible to inter-observer variation [21]. Notably, two hyposplenic patients with demonstrable HJBs had normal PRC values, which is unexpected as HJBs have generally only been reported when PRCs > 8% [20], highlighting the potential for variation using this technique. Collectively, our findings would argue against routine screening for hyposplenism in CD unless there is a specific clinical indication.

In contrast to prior studies examining spleen filtering capacity [11,12,55,56], we directly examined the impact of a GFD on spleen immune function. There was a large degree of inter-patient variability, and no clear trends of improvement after 6 months on a GFD were demonstrated. It is well established that mucosal healing rates are slow in adults with CD, and most have demonstratable enteropathy even after 6 months of a strict GFD [5]. Therefore, a follow-up considerably beyond 6 months and increasing cohort size may be necessary to reveal a beneficial effect of the GFD on spleen function. Nevertheless, as subtle defects are present even in CD patients treated for over 12 months, we suspect that not all patients will normalise on a GFD. Additionally, due to an increased awareness of CD and earlier diagnosis, our active CD cohort was much younger than our healthy and treated CD cohorts, which may impact their IgM memory B-cell response.

We did observe that CD patients diagnosed after 50 years had a higher frequency of hyposplenism than those diagnosed earlier in life, suggesting that prolonged gluten exposure prior to diagnosis was a factor. This finding is important as studies have noted an increasing incidence of CD in elderly people [61,62]. There was a positive correlation between years on a GFD and TD IgM memory B-cell response, and pneumococcal vaccine responses were lower in patients diagnosed after 50 years. These findings underscore the importance of an early diagnosis of CD and offering pneumococcal vaccination to CD patients ideally before the age of 50.

## 5. Conclusions

This is the first randomised study comparing pneumococcal vaccine responses in vaccine-naïve CD patients. We assessed protective immunity based on serotype-specific antibody levels and OPA, the latter considered the gold standard functional correlate of protection [63]. While both vaccines were immunogenic in CD, 13vPCV induced sustained protection for clinically important pneumococcal serotypes compared with 23vPPV. This is consistent with data showing that 13vPCV induces better protection in splenectomised patients [37,64]. 13vPCV also induces stronger memory responses than 23vPPV [65], although this was not assessed in the current study. Given the robust population data highlighting the increased risk of serious pneumococcal infections in CD and our findings demonstrating a high frequency of functional B-cell defects, there is a strong case to support CD being included in the list of diseases at high risk of pneumococcus, independent of the presence of overt hyposplenism. This classification is important to align the CD vaccination protocol to that given for asplenic/hyposplenic patients, and is relevant for national immunisation programs that provide funded vaccinations to at-risk groups.

Pneumococcal vaccination programs are evolving in many countries with the recent availability of the higher valency conjugate vaccines 15vPCV and 20vPCV [64]. Consequently, vaccination schedules for asplenia/hyposplenism have been adapted to involve priming with one dose of a conjugate vaccine (13vPCV, 15vPCV, or 20vPCV) followed by two polysaccharide boosters to broaden the serotypes protected against [14,66]. Although pneumococcal vaccination with 13vPCV is now routine in children in most high-income countries [64], there remains a large pool of pneumococcal-vaccine-naïve adult CD patients who would benefit from vaccination.

## Figures and Tables

**Table 1:** Demographics and hyposplenism frequency in patient cohorts.

	Healthy (n = 15)	Treated CD (n = 59)	Active CD (n = 6)	Splenectomised (SP; n = 11)
Age in years mean (range)	33 (22–63)	53 (22–74)	32 (28–36)	57 (39–65)
Sex (F; M)	9; 6	43; 15	3; 3	8; 3
HJBs Present	1/15	2/59	1/6	10/11
PRCs > 4% (median)	0/15 (0%)	2/59 (0.4%)	0/6 (0.2%)	10/11 (32.45%)
Hyposplenism (%)	1/15 (6.7%)	3/59 (5.1%)	1/6 (17%)	10/11 (91%)

CD, coeliac disease; HJBs, Howell–Jolly bodies; PRCs, pitted red cells; F, female; M, male.

**Table 2:** Demographics and hyposplenism metrics in treated CD subgroups.

	Complicated CD (n = 12)	Uncomplicated CD (n = 46)	CD Diagnosis < 50 Years (n = 40)	CD Diagnosis ≥ 50 Years (n = 18)	Uncomplicated CD and Diagnosis < 50 Years (n = 35)
Age in years (mean, range)	58, 28–74	59, 22–71	49, 22–71	65, 58–74	50, 22–71
PRCs median, range %	0, 0–41.3	0.4, 0–5.6	0.4, 0–1.9	0.4, 0–41.3	0.4, 0–1.9
Spleen volume median, range (cm^3^)	198, 33–342	201, 25–485	213, 76–485	183, 25–360	207, 76–485
Hyposplenism	1/12 (8.3%)	2/46 (4.3%)	1/40 (2.5%)	2/18 (11.1%)	1/35 (2.9%)

**Table 3:** Hyposplenism readouts in hyposplenic treated CD patients.

	HJBs	PRCs	Comorbidities	Spleen Size	TD Response	TI Response
TC02	Neg	Pos	Diagnosed > 50 years	Normal	Low	Low
TC03	Pos	Neg	None	Normal	Low	Low
TC53	Pos	Pos	Diagnosed > 50 years, autoimmune disease	Small	Normal	Low
TC23	Neg	Neg	Diagnosed > 50 years	Small	Low	Low
TC33	Neg	Neg	None	Small	Low	Normal
TC39	Neg	Neg	Diagnosed > 50 years	Small	Low	Normal

## Data Availability

The data that support the findings of this study are available from the corresponding author upon reasonable request.

## Supplementary Materials

The following supporting information can be downloaded at: https://www.mdpi.com/article/10.3390/vaccines12020214/s1, Figure S1: Division kinetics following TI stimulation of IgM memory B cells; Table S1: Participant clinical information; Table S2: Pneumococcal vaccine adverse reactions.
