# Supplementary material for: Impaired IgM Memory B Cell Function Is Common in Coeliac Disease but Conjugate Pneumococcal Vaccination Induces Robust Protective Immunity
Source: Vaccines (Basel). 2024 Feb 19;12(2):214. doi: 10.3390/vaccines12020214 (PMC10891918; doi:10.3390/vaccines12020214)
Supplement: Supplementary file 1 [file vaccines-12-00214-s001.zip › vaccines-2871051-supplementary.pdf]

Supplementary Table S1. Participant clinical information.

ID[A]	Year CD diagnosed[B]	Yrs on GFD	Sex	Age (yr)	Age at CD diagnosis (yr)[C]	HLA-DQ[D]	Medical History[E]	CD Serology[F]	HJB[G]	PRC %[H]	Complement studies[I]	Spleen volume (cm^3)	Vax[J]
TC01	2007	13	F	61	49	2.5 / 8	Nil of note	NAD[K]	Neg	0.8	NAD	158	PPV
TC02	2010	10	M	66	**57**	2.5 Het	Previous pneumococcal vax	NAD	Neg	5.6	NAD	151	
TC03	1966	54	M	56	3	2.5 / 8	Thyroid nodules	NAD	Pos	1	NAD	251	PCV
TC04	2015	5	F	44	40	2.5 Het	Ductal carcinoma in situ, Grave's disease	NAD	Neg	0.4	NAD	228	PCV
TC05	2017	3	F	26	24	8 Het	Nil of note	TTG 31	Neg	0	NAD	223	
TC06	1997	23	M	68	46	2.2	Young's Syndrome, previous pneumococcal vax	NAD	Neg	0	NAD	164	
TC07	2017	3	F	26	24	2.5 Het	Nil of note	NAD	Neg	0.8	C4 0.13 g/L	270	
TC08	2010	10	F	61	**52**	2.5/ 8	Nil of note	NAD	Neg	0	C3 0.8 g/L, C4 0.14 g/L	75	PCV
TC09	2002	18	F	65	48	2.5 Het	Nil of note	NAD	Neg	0.8	NAD	90	PCV
TC10	2015	5	F	70	**66**	2.5 Het	Previous pneumococcal vax, Hashimoto's (2012)	NAD	Neg	0	NAD	198	
TC11	2017	3	F	28	26	2.5 Homo	Nil of note	NAD	Neg	0.7	NAD		PPV
TC12	2009	11	F	62	**52**	2.5 Homo	Nil of note	NAD	Neg	0.8	NAD	360	PPV
TC13	2019	1	F	22	22	2.5 Het	Nil of note	NAD	Neg	0.3	NAD	330	
TC14	2013	7	M	58	**52**	2.5 / 2.2	Hashimoto's (2016)	NAD	Neg	1.2	NAD	183	PCV

TC15	2013	6	M	30	25	2.2 / 8	Pneumonia as a child	NAD	Neg	0	NAD	350	PPV
TC17	2008	12	F	35	24	2.5 Het	Nil of note	NAD	Neg	1.6	NAD	164	PPV
TC18	2009	11	F	51	41	2.5 Het	Nil of note	NAD	Neg	0	NAD	260	PCV
TC19	2012	8	F	42	35	2.5 / 8	Nil of note	NAD	Neg	0.4	NAD	121	PPV
TC20	2016	4	F	28	25	2.5 / 8	Nil of note	NAD	Neg	1.2	C3 1.98 g/L	213	PPV
TC21	2016	4	F	68	**65**	2.5 Het	Nil of note	NAD	Neg	2	NAD	180	PPV
TC22	1964	29	M	58	29	2.5 Het	Nil of note	DGP 71	Neg	0	NAD	107	PPV
TC23	2005	16	M	66	**50**	2.5 Het	Nil of note	NAD	Neg	2.4	NAD	51	PCV
TC24	2018	3	F	51	48	8	Vitiligo	NAD	Neg	0.4	NAD	234	
TC25	2013	8	F	52	44	2.5 Het	Nil of note	NAD	Neg	0.4	NAD	201	
TC26	2000	21	F	64	43	2.5	Pneumonia 2019	NAD	Neg	0	NAD	240	PCV
TC27	1984	37	F	67	30	2.5 Het	Nil of note	NAD	Neg	0	NAD	189	
TC28	2019	2	F	31	29	2.5 Homo	Previous pneumococcal vax	NAD	Neg	0	NAD	163	
TC29	2017	4	F	47	43	2.5 Het	RCD	TTG 35	Neg	0.4	NAD	175	PPV
TC30	2004	17	M	57	40	?2.2/8	Nil of note	TTG 61	Neg	0	NAD	251	PPV
TC31	2015	6	F	41	35	2.5 Het	Nil of note	NAD	Neg	0	C3 0.79 g/L	128	PCV
TC32	2013	8	M	63	**55**	2.5 Het	Nil of note	NAD	Neg	0	C3 0.74 g/L, C4 0.12 g/L	291	PPV
TC33	1997	24	M	70	46	2.5 Het	Type 2 DM, Previous pneumococcal vax (2018)	NAD	Neg	0	NAD	88	

TC34	2019	2	M	71	**69**	2.2/8	Idiopathic thrombocytopaenia/ RCD	Not Done		0	Not done	Not done	
TC35	2001	20	F	65	45	2.5 Het	Nil of note	NAD	Neg	0.4	NAD	76	PCV
TC36	2010	11	F	62	**51**	2.5 Het	Neutropenia	NAD	Neg	0.4	NAD	305	PPV
TC37	2019	2	F	68	**66**	2.5 Het	Leiomyosarcoma 2007, Pneumonia 2018 (hospitalised), previous pneumococcal vax 2018	NAD	Neg	0.4	NAD	196	
TC38	2012	9	F	64	**55**	2.5	Hashimoto's	NAD	Neg	0.8	NAD	94	PCV
TC39	2016	5	M	67	**62**	2.5 Het	Previous pneumococcal vax	TTG 55	Neg	0.4	NAD	25	
TC40	2007	14	F	37	23	8	Narcolepsy	NAD	Neg	0	NAD	485	PCV
TC41			F	59		2.5 Het	Hemochromatosis	NAD	Neg	0	NAD		
TC42	2009	12	F	65	**53**	2.5 Het	Previous pneumococcal vax	NAD	Neg	0.4	C4 0.12 g/L	210	
TC43	2014	7	F	32	25	2.5 Homo	Nil of Note	NAD	Neg	0.4	C3 0.70 g/L, C4 0.12 g/L	265	
TC44	2010	11	F	57	46	2.5 Het	Rheumatoid Arthritis	NAD	Neg	0	NAD	Not done	PCV
TC45	1975	46	M	65	19	2.5 Het	Nil of Note	NAD	Neg	0.4	NAD	Not done	PPV
TC46	2019	2	F	59	**57**	2.5 Het	Nil of Note	NAD	Neg	0	NAD	296	PCV
TC47	2012	9	F	49	40	2.5 Het	Nil of Note	NAD	Neg	0.4	C3 0.71 g/L, C4 0.14 g/L	Not done	PCV
TC48	2015	6	F	27	21	2.5 Het	Proctitis	NAD	Neg	0.4	NAD	Not done	PCV

TC49	2013	8	F	28	20	2.5 Homo	Hashimoto's disease (2019)	NAD	Neg	0	C4 0.14 g/L	247	
TC50	2002	19	F	25	6	2.5 Het	Nil of Note	NAD	Neg	1.9	NAD	373	
TC51	2010	11	F	41	30	2.5 Het	Psoriatic Arthritis (2013)	TTG 25	Neg	1.4	NAD	342	PPV
TC52	2012	9	F	43	34	2.5/2.2	Nil of Note	NAD	Neg	1.6	C4 0.13 g/L	Not done	PPV
TC53	2020	1	F	66	**65**	2 / 8	Kidney donor, pneumonia 2018, Rheumatoid Arthritis (2019)	Pos	Pos	41.3	NAD	33	
TC54	2017	4	M	59	**55**	2.5 / 2.2	Neutropoenia	NAD	Neg	0.8	NAD	Not done	PPV
TC55	1999	22	F	61	39	2.5 Homo	Pneumonia 2015	TTG 33	Neg	1.2	NAD	Not done	PPV
TC56	2008	13	F	34	21	2.5/ 8	Thyroid cyst	NAD	Neg	0	NAD	Not done	PPV
TC57	2012	8	F	74	**66**	2.5 Het	Refractory coeliac disease		Neg	0.4	NAD	Not done	
TC58			M	71		2.5	Hemochromatosis, previous pneumococcal vax	NAD	Neg	0.4	NAD	Not done	
TC59	2003	18	F	63	45		Fibromyalgia, asthma, TIA	NAD	Neg	0.8	NAD	Not done	
TC60	2004	17	F	51	34	2.5/2	Hypertension, schizophrenia	TTG 21	Neg		NAD	Not done	
AC01	Nov 2020	<1	F	28	28	8	Crohn's disease (2017)	**TTG 965, DGP 82**	Neg	0.4 (BS); 0.4 (FU)	NAD	305	N/A
AC02	Dec 2020	<1	F	36	36	2.5 Het	Nil of note	**TTG 181, DGP 36**	Pos	1.1 (BS)	NAD	121	N/A
AC03	Dec 2020	<1	F	33	33	2.5 Het	Gestational Diabetes	**TTG 42, DGP 138**	Neg	0 (BS); 0 (FU)	C3 0.76g/L	275	N/A

AC04	May 2021	<1	M	30	30	8	Nil of note	**TTG 660, DGP 41**	Neg	0 (BS); 0.4 (FU)	NAD	Not done	N/A
AC05	May 2021	<1	M	28	28	2.5 Homo	Palmoplantar keratoderma	**TTG 884, DGP 551**	Neg	0.4 (BS); 0 (FU)	C4 0.13g/L	Not done	N/A
AC06	Sep 2021	<1	M	34	34	2.5 Het	Nil of note	**TTG 758, DGP 115**	Neg	0 (BS); 0 (FU)			N/A
SP01	N/A	N/A	F	62	N/A	2.5 Homo	Coeliac disease (2006), splenectomy 1974 for idiopathic thrombocytopaenia	NAD	Pos	34.9	NAD	N/A	N/A
SP02	N/A	N/A	F	63	N/A	Neg	Splenectomy 2013 for pseudomyxoma peritonei	NAD	Pos	39.9	NAD	N/A	N/A
SP03	N/A	N/A	F	65	N/A	2.2/8	1997 splenectomy for idiopathic thrombocytopaenia	NAD	**Pos**	39.8	NAD	N/A	N/A
SP04	N/A	N/A	F	47	N/A	2.2	2017 splenectomy for idiopathic thrombocytopaenia, partial nephrectomy, renal cell carcinoma, hypertension	NAD	**Pos**	6.5	NAD	N/A	N/A
SP05	N/A	N/A	F	55	N/A	2.2	2006 splenectomy for incidental benign tumour	NAD	**Pos**	42.6	NAD	N/A	N/A
SP06	N/A	N/A	M	58	N/A		1979 splenectomy for Hodgkin's Lymphoma	TTG 336	**Pos**		NAD	N/A	N/A
SP07	N/A	N/A	F	63	N/A	2.5 het	1998 – Motor vehicle accident, coeliac disease (2016)	NAD	Neg	3.1	TTG 336	N/A	N/A
SP08	N/A	N/A	M	39	N/A	2.5 het	2008 splenectomy for idiopathic	NAD	**Pos**	25.9	NAD	N/A	N/A

							thrombocytopaenia, hypothyroidism						
SP09	N/A	N/A	F	64	N/A	2..5/8	1975 – splenectomy for motor vehicle accident	NAD	**Pos**	40.4	NAD	N/A	N/A
SP10	N/A	N/A	M	51	N/A	2.2	1982 – Splenectomy for spherocytosis, Ewing's sarcoma, hypertension, renal calculi	NAD	**Pos**	30.0	NAD	N/A	N/A
SP11	N/A	N/A	F	62	N/A	2.5/7	1982 – splenectomy for spherocytosis, hypertension		**Pos**	25.5		N/A	N/A
HV01	N/A	N/A	M	26		2.5 Het	Nil of Note	NAD	Neg	0	NAD	N/A	N/A
HV02	N/A	N/A	M	21		2.2/7	Nil of Note	NAD	Neg	0.4	NAD	N/A	N/A
HV03	N/A	N/A	M	24		Neg	Nil of Note	NAD	Neg	0	NAD	N/A	N/A
HV04	N/A	N/A	M	30		Neg	Nil of Note	NAD	Neg	0	NAD	N/A	N/A
HV05	N/A	N/A	F	25		Neg	Pneumonia as teenager	NAD	Neg	0.4	NAD	N/A	N/A
HV06	N/A	N/A	F	52		Neg	Nil of Note	NAD	Neg	0	NAD	N/A	N/A
HV07	N/A	N/A	F	24		8 Het	Nil of Note	NAD	Neg	0	NAD	N/A	N/A
HV08	N/A	N/A	M	24			Nil of Note	NAD	Neg	1.2	NAD	N/A	N/A
HV09	N/A	N/A	F	45		Neg	Nil of Note	NAD	Neg	2.0	NAD	N/A	N/A
HV10	N/A	N/A	F	25		Neg	Nil of Note	NAD	Neg	0.0	NAD	N/A	N/A
HV11	N/A	N/A	F	28		Neg	Nil of Note	NAD	Neg	0.0	NAD	N/A	N/A
HV12	N/A	N/A	M	25		2.2	Elevated IgE	NAD	Neg	0.4	NAD	N/A	N/A
HV13	N/A	N/A	F	62		2.5/2.2	Nil of Note	NAD	Pos	1.6	NAD	N/A	N/A
HV14	N/A	N/A	F	51		2.5 Het	Pituitary adenoma	NAD	Neg	0.8	NAD	N/A	N/A
HV15	N/A	N/A	F	43		2.5 Het	Nil of Note	NAD	Neg	0	NAD	N/A	N/A

[A] Table shading indicate the following cohorts: dark blue = treated CD (TC); light blue = active CD (AC); purple = splenectomised (SP); green = healthy volunteer (HV).

[B] Dx; diagnosis

[C] Bold; patients diagnosed >50 years of age

[D] HLA; human leukocyte antigen, Het; heterozygous, Homo; homozygous

[E] Pink shading indicates patients with autoimmune disease or refractory coeliac disease (RCD) categorised as complicated CD

[F] CD serology normal ranges: tissue transglutaminase (TTG) <20 and deamidated gliadin peptide (DGP) <20.

[G] HJB; Howell-Jolly bodies

[H] PRC: Pitted red cell percentage

[I] Complement normal ranges: C3, 0.8-1.6g/L; C4, 0.16-0.48g/L

[J] Vax: indicates participants who received either 23vPPV or PCV13 as part of this study.

[K] NAD: no abnormalities detected

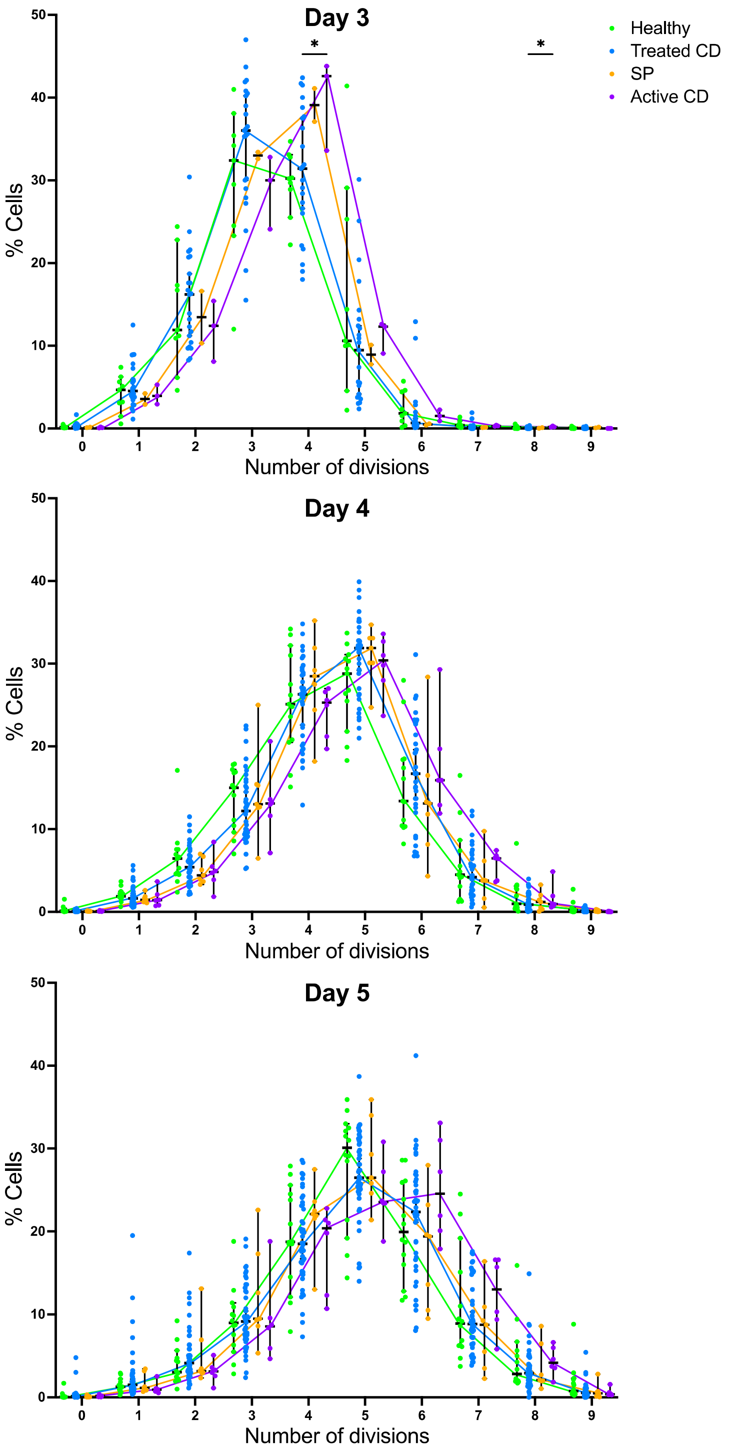

Supplementary Figure S1. Division kinetics following TI-stimulation of IgM memory B cells. The proportion of TI-stimulated cells within each division are shown at day 3, 4 and 5. Data are shown for each participant group; healthy, treated CD, splenectomised (SP) and active CD. Frequencies are compared by Mann-Whitney U-tests (two-tailed). Horizontal lines on graphs indicate median and error bars indicate 95% confidence intervals. * P<0.05.

Supplementary Table S2. Pneumococcal vaccine adverse reactions.

ID	Vaccine	Reaction Y/N	Comments
TC01	23vPPV	N	
TC03	PCV13	Y	Sore arm – injection site.
TC04	PCV13	N	
TC08	PCV13	Y	Sore arm – injection site.
TC09	PCV13	N	
TC11	23vPPV	N	
TC12	23vPPV	N	
TC14	PCV13	N	
TC15	23vPPV	N	
TC17	23vPPV	Y	Sore arm – injection site.
TC18	PCV13	N	
TC19	23vPPV	Y	Sore arm – injection site.
TC20	23vPPV	Y	Sore arm – injection site.
TC21	23vPPV	N	
TC22	23vPPV	N	
TC23	PCV13	N	
TC26	PCV13	Y	Tiredness, headaches, flu-like symptoms. Settled after one week.
TC29	23vPPV	N	
TC30	23vPPV	N	
TC31	PCV13	N	
TC32	23vPPV	N	
TC35	PCV13	N	
TC36	23vPPV	Y	Shoulder bursitis attributed to a high (bursal) injection. Settled with conservative care.
TC38	PCV13	Y	Sore arm – injection site.
TC40	PCV13	Y	Sore arm – injection site.
TC44	PCV13	N	
TC45	23vPPV	N	
TC46	PCV13	N	
TC47	PCV13	Y	Sore arm – injection site.
TC48	PCV13	N	
TC51	23vPPV	Y	Sore arm – injection site.
TC52	23vPPV	N	
TC54	23vPPV	N	
TC55	23vPPV	N	
TC56	23vPPV	N	
